# Consolidation, Systematic Appraisal and Comparison of Guideline Recommendations Regarding Management of Chronic Pain: Protocol for a Digital Chronic Pain Recommendation Map

**DOI:** 10.1002/gin2.70033

**Published:** 2025-08-11

**Authors:** Andrea J. Darzi, Kian Torabiardakani, Gonzalo Bravo‐Soto, Daniela Montalva‐Romero, Rachel J. Couban, Lynn Cooper, Stacey Ritz, Jaris Swidrovich, Ivan D. Florez, Vivian Welch, Gordon H. Guyatt, Holger J. Schünemann, Jason W. Busse, on behalf of the Chronic Pain RecMap Collaborative, Ahmad Firas Khalid, Abhimanyu Sud, Alfonso Iorio, Anaïs Lacasse, Andrew Thomas, Artur Nowak, Bart Dietl, Behnam Sadeghirad, Cyd Courchesne, Dena Zeraatkar, Edoardo Aromataris, Elaine Harrow, Farid Foroutan, Fei Yutong, Gabriel Rada, Glen Hazlewood, Hugo Massé‐Alarie, Jennifer Petkovic, Justyna Litynska, Keven Phinney, Lawrence Mbuagbaw, Li Wang, Lisa Graves, Luc J. Hébert, Marc‐André Blanchette, Peter Tugwell, Rebecca Morgan, Jean‐Sebastien Roy, Thomas Piggott, Trevor Jenvenne, Yaolong Chen, Ying Zhang, Zachary Munn, Zoe Jordan, Rana Yamout

**Affiliations:** ^1^ Department of Health Research Methods, Evidence, and Impact McMaster University Hamilton Ontario Canada; ^2^ Department of Anesthesia McMaster University Hamilton Ontario Canada; ^3^ Michael G. DeGroote National Pain Center McMaster University Hamilton Ontario Canada; ^4^ Department of Pathology & Molecular Medicine McMaster University Hamilton Ontario Canada; ^5^ Leslie Dan Faculty of Pharmacy University of Toronto Toronto Ontario Canada; ^6^ Department of Pediatrics University of Antioquia Medellin Colombia; ^7^ Pediatric Intensive Care Unit Clınica Las Americas‐AUNA Medellin Colombia; ^8^ School of Rehabilitation Science McMaster University Hamilton Ontario Canada; ^9^ Bruyère Health Research Institute University of Ottawa Ottawa Ontario Canada; ^10^ School of Epidemiology and Public Health University of Ottawa Ottawa Ontario Canada; ^11^ Clinical Epidemiology and Research Center (CERC) Humanitas University & Humanitas Research Hospital Milan Italy; ^12^ Ottawa Hospital Research Institute Ottawa Ontario Canada; ^13^ Department of Family and Community Medicine University of Toronto Toronto Ontario Canada; ^14^ Département des sciences de la santé Université du Québec en Abitibi‐Témiscamingue (UQAT) Rouyn‐Noranda Québec Canada; ^15^ Chronic Pain Centre of Excellence (CPCoE) Hamilton Ontario Canada; ^16^ Evidence Prime Kraków Poland; ^17^ Health Professionals Division Veterans Affairs Canada Ottawa Ontario Canada; ^18^ Faculty of Health and Medical Sciences The University of Adelaide Adelaide South Australia Australia; ^19^ Guideline International Network; ^20^ GRADE center Beijing University of Chinese Medicine Beijing China; ^21^ Epistemonikos Foundation Santiago Chile; ^22^ Department of Medicine, Cumming School of Medicine University of Calgary Calgary Alberta Canada; ^23^ Faculty of Medicine Université Laval Québec Québec Canada; ^24^ Bruyere Research Institute University of Ottawa Ottawa Ontario Canada; ^25^ Family and Community Medicine Western Michigan University Homer Stryker M.D. School of Medicine Kalamazoo Michigan USA; ^26^ School of Rehabilitation Sciences, Faculty of Medicine Université Laval Québec City Quebec Canada; ^27^ Department of Chiropractic Université du Québec à Trois‐Rivières Trois‐Rivières Quebec Canada; ^28^ University of Ottawa, Faculty of Medicine and Bruyère Health Research Institute and Ottawa Hospital Research Institute Ottawa Ontario Canada; ^29^ Dominion Command Sports Committee The Royal Canadian Legion Ottawa Ontario Canada; ^30^ Evidence‐based Medicine Center, School of Basic Medical Sciences Lanzhou University Lanzhou China; ^31^ School of Traditional Chinese Medicine Beijing University of Chinese Medicine Beijing China; ^32^ Health Evidence Synthesis, Recommendations and Impact (HESRI), School of Public Health, Faculty of Health and Medical Science The University of Adelaide Adelaide South Australia Australia; ^33^ JBI, Faculty of Health and Medical Sciences The University of Adelaide Adelaide South Australia Australia; ^34^ Division of General Internal Medicine and Geriatrics and Department of Internal Medicine American University of Beirut Beirut Lebanon

## Abstract

**Introduction:**

Chronic pain affects 1 in 5 adults and children globally; however, management remains highly variable and low value care is common. Inconsistent recommendations among clinical practice guidelines for management of chronic pain contribute to suboptimal patient management. We will develop and disseminate a living digital chronic pain recommendation map (e‐Chronic Pain RecMap) to identify trustworthy recommendations in three high priority areas, (1) opioids, (2) cannabis for medical purposes, and (3) spine‐related interventional procedures for chronic pain.

**Methods:**

The project comprises three phases. A planning phase to engage a diverse group of interest holders to co‐design a team structure with our knowledge users to ensure an efficient and effective workflow. Through a search of electronic databases and a manual search of international websites, a development phase to systematically identify relevant guidelines published in any language since 2019. In this development phase, we will update our searches every 6 months. Also, we will appraise the reporting quality of eligible guidelines using the AGREE‐II instrument, followed by appraisal of recommendation‐level quality using AGREE‐REX for guidelines meeting a defined threshold. In a next step, we will extract data from guidelines including relevant equity information using infrastructure in GRADEPro, and explore divergence and compare recommendations answering the same guideline question. Lastly, we will develop plain language summaries of trustworthy recommendations and decision aids to support patient‐physician decision making, and translate the platform to French, Spanish, and Mandarin. Finally, a mobilization phase in which, with our interest holders, we will co‐create strategies to disseminate the RecMap to relevant target users.

**Discussion:**

The Chronic Pain RecMap will enhance use of trustworthy guideline recommendations by people living with chronic pain, clinicians, and decision makers. Successful uptake will optimize evidence‐based pain management.

## Summary

What is already known on this topic?

- Chronic pain is a common condition affecting 1 in 5 adults and children globally.
- Hundreds of clinical practice guidelines have been developed to optimize practice but vary widely in quality and often present inconsistent recommendations.
- There is an urgent need to increase chronic pain literacy across healthcare systems and among the public at large, and improve concordance between evidence and practice.

What this study adds?

- A plan to co‐design and develop a living digital chronic pain recommendation map.
- Methods to consolidate, systematically appraise and compare guideline recommendations in three high‐priority areas for chronic pain including opioids, cannabis and spine‐rated interventional procedures.
- A plan to translate and mobilize knowledge from the RecMap based on co‐creation with national and international collaborators and interest holders.

## 1 Background

Chronic pain is a major public health concern, affecting approximately 20% of individuals worldwide and resulting in substantial socioeconomic burden [1]. In Canada, around 7.6 million people experience chronic pain, including one in five children and young adults and one in three adults over age 65 [1]. Aging and population growth will result in an estimated 9 million Canadians will be affected by chronic pain by 2030, accounting for up to $23.4 billion in direct and $31.5 billion in indirect annual costs [1]. Studies have reported comparable prevalence rates in the United States and Europe, underscoring the impact of chronic pain globally [2]. Moreover, healthcare costs for management of chronic pain have increased substantially over time without evidence of improved outcomes for patients [3, 4]. There are many reasons for this disconnect, including overuse of opioids [5] and widespread delivery of invasive procedures that have shown a net negative impact [6, 7].

There is thus an urgent need to increase chronic pain literacy across healthcare systems and among the public at large, and improve concordance between evidence and practice [1]. The Knowledge to Action framework describes health care guidelines as a key tool to promote evidence‐based patient care [8]. Guideline recommendations aim to support clinicians, patients, and their caregivers in navigating uncertainty regarding optimal choices for pain management.

Current clinical practice guidelines for chronic pain often offer divergent recommendations. For example, the 2022 US Department of Defense and US Department of Veterans Affairs' guideline made a strong recommendation against use of opioids for chronic pain [9], whereas the 2022 US Centers for Disease Control and Prevention (CDC) guideline made a strong recommendation that ‘[clinicians should] consider initiating opioid therapy if expected benefits for pain and function are anticipated to outweigh risks to the patient’ [10]. Reasons for divergent recommendations include variability in the evidence considered, poor quality of guidelines, unmanaged conflicts of interest among guideline panel members, and failure to consider patients' values and preferences [11]. The technical and time‐consuming considerations required to establish which recommendations are trustworthy requires efforts unrealistic for individual users. Thus, there is a need to develop and make accessible to users a systematic appraisal and evaluation of the trustworthiness of chronic pain guidelines recommendations [11].

Recommendation mapping, which may enhance access to trustworthy guidance and inform clinical, public health, and health systems planning and decision‐making, offers a promising solution [12]. This approach visually categorizes guideline recommendations by populations and interventions, facilitating the development of digital platforms like the Recommendation Map (RecMap). A RecMap organizes and maps guideline recommendations, displaying critical information such as the evidence base, trustworthiness, and development process [12, 13, 14]. It also enables users to identify knowledge gaps and clusters by searching research priorities or using filters to highlight equity‐sensitive recommendations based on the PROGRESS PLUS framework (Place of residence, Race/ethnicity/culture/language, Occupation, Gender, Religion, Education, Socioeconomic status, Social capital, and ‘Plus’ that includes other context‐specific factors). These features of the RecMap allow users to identify trustworthy recommendations that best fit their need and context [15, 16]. RecMaps also allow guideline developers to adapt existing recommendations to different settings by accessing, when available, underlying evidence and tailoring considerations such as values, feasibility, acceptability, equity, or cost [15].

We propose developing a digital Chronic Pain RecMap that will initially focus on three interventions: opioids, cannabis, and common spine‐related interventional procedures. We will create and mobilize a freely accessible RecMap to provide users with trustworthy recommendations, highlight gaps in guidance, and facilitate evidence‐based patient care.

## 2 Objectives

To develop and mobilize a freely accessible digital interactive Chronic Pain RecMap that will identify the trustworthiness of guideline recommendations addressing the use of opioids, cannabis, and spine‐related interventional procedures for chronic pain.

## 3 Methods

We co‐developed our approach and multi‐phase methods with our knowledge users, grounding them in evidence mapping methodology and the blueprints and methods from previous recommendation mapping efforts [12, 15]. This project will be conducted in three phases including (1) planning phase and chronic pain RecMap working groups, (2) RecMap development and integrated early knowledge mobilization phase and (3) end of project knowledge mobilization phase. We describe the details of each phase below.

### 3.1 Phase 1: Planning Phase and Chronic Pain RecMap Working Groups

**Figure 1:**
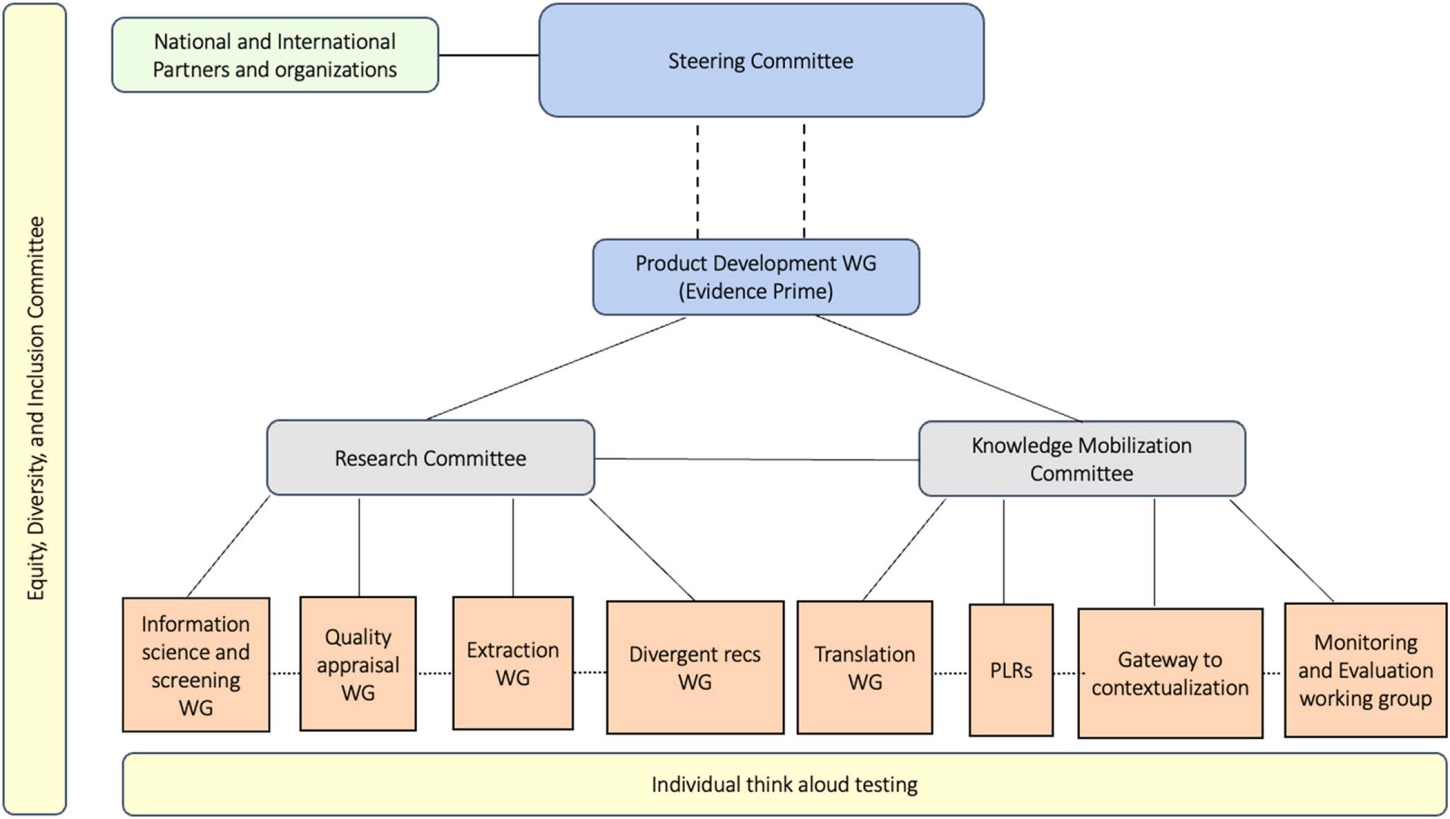
Structure of team and workflow for development and maintenance of the Chronic Pain RecMap.

We co‐designed the structure of our team with knowledge users to ensure an efficient, scientifically rigorous, and equity‐informed workflow (Figure 1). The eight‐member steering committee (AJD, JWB, GHG, HJS, SR, VW, LC, and JS) will provide strategic oversight for methodological and managerial decisions. This committee will guide the activities of all RecMap working groups, ensuring alignment with the project's objectives and adherence to best practices. A diverse group of provincial, national, and international interest holders (detailed in Table 1) will engage in structured consultations with the steering committee throughout the RecMap development and mobilization process. The Evidence Prime [17] working group, composed of product designers and software developers, will be responsible for designing the RecMap platform to include relevant variables, data, program filters, and search functionalities. The research committee (AJD, KT, GBS, and DMR) will train and oversee teams of reviewers to conduct systematic screening, appraisal of guidelines and recommendations, and extraction of relevant data to populate the RecMap (Figure 1). This process will follow pre‐defined eligibility criteria and standardized methods to ensure the reliability and validity of included information.

**Table 1:** Interest holders.

Organization Name
Health Canada
Canadian Medical Association
Quebec Pain Research Network (QPRN)
The Association of Faculties of Medicine of Canada (AFMC) response to opioid crisis
Pain BC
Veterans Affairs Canada
Federation of Medical Regulatory Authorities of Canada
Canadian Injured Workers Alliance
Effective Prescribing of Opioids for Chronic Pain (EPOCH)
Chronic Pain Network
Michael G. DeGroote Institute for Pain Research & Care (IPRC)
The Canadian Rheumatology Association (CRA)
Michael G. DeGroote National Pain Centre (MGD NPC)
Canada Life
Cochrane Canada/GRADE center
Cochrane Methods Equity
Chinese GRADE center, Lanzhou University
Beijing GRADE center
Cochrane China
World Health Organization (WHO) Collaborating Centre for Guideline Implementation and Knowledge Translation in China
Guideline international network (GIN)
Cochrane Colombia
American Academy of Family Physicians (AAFP)
Cochrane Ibéroamerica
JBI (previously known as the Joanna Briggs Institute)
The Appraisal of Guidelines for Research and Evaluation (AGREE) enterprise
Cochrane Insurance Medicine
Epistemonikos
Evidence Prime

The knowledge mobilization (KM) committee, which will include KM experts and interest holders from supporting organizations, will be responsible to engage with interest holders to prioritize and co‐develop strategies to mobilize the RecMap including unidirectional, bidirectional, engagement, and implementation activities (examples are presented in Table 2). The equity, diversity, and inclusion (EDI) committee (AJD, JS, VW, SR), will ensure that EDI, including sex and gender considerations, are integrated throughout the planning, development, and dissemination phases. Specific tasks include ensuring equitable team representation, expanding partnerships with marginalized communities, and fostering inclusive and collaborative environments. The EDI committee will also ensure patient collaborators are compensated fairly in accordance with Canadian Institutes of Health Research (CIHR) Strategy for Patient‐Oriented Research (SPOR) guidelines and will adopt inclusive recruitment strategies to promote diversity [18]. Also, the EDI committee will train teams to systematically extract equity relevant data from guidelines and evaluate whether EDI considerations were explicitly addressed in the development of recommendations.

**Table 2:** Examples of knowledge mobilization activities.

KM activities	Examples	Knowledge user audiences	Monitoring and evaluation
Unidirectional activities	Outreach campaigns, conference presentations, publications webinars/presentations to professional associations/networks, educational presentations in academic institutions (Medical school)	Clinicians/practitioners, researchers, and funders	Number of publications, citations, presentations, number of invitees who participated, etc.
	Dissemination through media to promote the RecMap	The public (consumers) (e.g., media, patients)	Number of views, shares, engagement
	Translation of the RecMap into French, Spanish and Mandarin	The public (consumers) (e.g., media, patients), Clinicians/Practitioners, Policymakers and health care managers, researchers)	Google analytics, findings from focus group discussions or surveys with the target knowledge users
Bidirectional activities	Workshops, deliberative dialogues, focus groups/interviews, consultations	Policymakers/decision makers, clinicians and other health care professionals, patients, researchers, and funders	Number of meetings/workshops; number of policy makers involved
Engagement activities	Converting standard language recommendations (SLR) to plain language recommendations (PLR) to enhance usability	The public (consumers) (e.g., media, patients)	Number of recommendations converted, traffic on RecMap to access PLRs, findings from focus group discussions with the target knowledge users
	Contextualization (adoption/adaptation of recommendations)	Clinician/practitioners, researchers (individual or professional health associations or organizations)	Case studies, guidelines in different contexts based on RecMap
Implementation activities	Integration into educational modules to enhance usability	Clinicians/practitioners	Google analytics; survey with HHS health professionals
	Integration into electronic medical records to enhance accessibility and usability of the Map		

### 3.2 Phase Two: RecMap Development and Integrated Early Knowledge Mobilization

#### 3.2.1 Guideline Eligibility Criteria

We will include guidance documents that provide recommendations, including evidence‐based guidelines as defined by WHO [19] and position statements [20]. We will include documents published in any language addressing the use of opioids, cannabis, or spine‐related interventional procedures for chronic pain in adults or children. When multiple versions of a guideline exist, we will include only the most recent version. We will limit our search to the past 5 years (2019 to present).

#### 3.2.2 Guideline Searches and Sources

Our search will rely on bibliographic database searches, manual searches, and targeted contacts. First, an experienced librarian (RJC) will develop and run searches in Medline and EMBASE using a combination of MeSH terms and keywords approved by Peer Review of Electronic Search Strategies (PRESS) (see Appendix 1 for search strategy). Second, we will conduct a manual search of ECRI Clinical Guidelines [21], International Database of GRADE Guidelines [22], Guideline Central [23], Trip Database [24], and International Guidelines Library [25]. Third, we will conduct manual searches of guideline organization websites (e.g., National Institute for Health and Care Excellence (NICE), Scottish Intercollegiate Guidelines Network (SIGN), WHO, Centers for Disease Control and Prevention (CDC), Veterans Affairs and Department of Defense (VA/DoD)). In addition, we will search in Guidelines registries PREPARE and Guideline international network (GIN) registry [26]. We will update our searches every 6 months or as needed to ensure the RecMap is up to date.

#### 3.2.3 Guideline Citation Screening and Management

Pairs of reviewers will screen, independently, each title and abstract and all potentially eligible full texts. We will then transfer all eligible guidelines to a Google worksheet for critical appraisal. We will ensure identification of guideline references by having reviewers extract general information from every eligible guideline document (i.e., authors, title, publication date, database/website) and generate a unique record ID.

#### 3.2.4 Critical Appraisal and Data Extraction of Guidelines and Recommendations

Teams of two trained assessors will appraise guideline quality using the AGREE‐II tool [27], which evaluates six domains: scope and purpose, stakeholder involvement, rigor of development, clarity of presentation, applicability, and editorial independence. We will calculate domain scores using AGREE‐II formulas. We will subsequently appraise the quality of individual recommendations from guidelines meeting a defined threshold using the AGREE‐REX tool, which evaluates Clinical Credibility, Stakeholder Values and Preferences, and Implementability [28, 29]. Given the uncertainty regarding quality of chronic pain guidelines eligible for our review, we will not prespecify a threshold. Instead, we will first systematically appraise all eligible guidelines with AGREE‐II and map their domain scores overall and within specific clinical topics (opioids, cannabis, spine‐related interventions). The Steering Committee will then iteratively determine an inclusion threshold through consensus, balancing methodological rigor and adequate representation across clinically important areas.

In a next step, one reviewer will extract data from each guideline and its recommendations into a customized GRADEpro platform [30], with verification by a senior team member. With regard to equity relevant information, we will extract data based on sex, gender, and other PROGRESS‐Plus factors. This includes subgroup analyses or meta‐regressions examining benefits and harms separately by sex and gender, and recommendations explicitly informed by these analyses. We will document whether guidelines address population representation, baseline risk differences, intersectional impacts (e.g., overlapping effects of sex, gender, race, socioeconomic status), and barriers to intervention access along with associated implementation considerations. Extracted equity‐related data will be highlighted within the RecMap. When equity‐related evidence is limited or unreported, these gaps will be identified in the platform's research priorities section or alongside the quality assessment. Ultimately, this platform will systematically present guideline‐level data (e.g., source, search date) and recommendation‐level details, facilitating tailored searches, ongoing updates and future contextualization efforts [15].

#### 3.2.5 Presentation of the Digital Platform and Recommendations

Evidence Prime will develop interfaces and present information on the platform tailored to meet the needs of our three user groups [31]. To tailor the platform's interfaces and information presentation, Evidence Prime will create interactive RecMap mockups that we will use to first conduct focus groups with our interest holders to receive feedback. We will consider this feedback and make suitable revisions to the mockups. We will then conduct individual think aloud testing with members of the public, including researchers, health care providers, policymakers, patient partners and families to further test usability of the different interfaces of the platform. Finally, the steering committee and Evidence Prime will discuss findings from the usability assessments and agree on final content and visual updates of the RecMap.

To ensure accessibility for all users, the platform will adhere to Web Content Accessibility Guidelines (WCAG) [32]. This includes offering captions for video content and transcripts for audio content included in the platform; using sufficient color contrast between text and background to ensure readability for users with visual impairments or color blindness; and ensuring users can resize, using browser settings, text up to 200% without loss of functionality or content [32].

The platform will offer multiple viewing options including lists of relevant recommendations, a visual map, and a comparison tool for recommendations based on predetermined criteria. The map will present a consultation grid that visually presents guidelines, recommendations, or research priorities by topic area with interventions displayed in rows and the populations in columns. Programmed filters as well as a free text search function will enhance navigation. Users will have the option to access detailed information for each recommendation including the quality of the source guideline and the recommendation of interest as per AGREE‐II and AGREE‐REX assessments; the certainty of the evidence (very low, low, moderate, or high); direction (for or against); and strength of the recommendation (strong or conditional). We anticipate guidelines included in the RecMap may use various classification systems for rating the certainty of evidence and strength of recommendations (e.g., SIGN, Oxford CEBM, ESC). To facilitate consistent comparison across recommendations, we will systematically transform these ratings into GRADE categories using the structured framework proposed by Klugar et al. This structured approach will support consistent interpretation and comparison of recommendations across diverse guidelines [33]. To facilitate understanding and application, we will provide additional resources such as evidence summaries, infographics, and decision aids [15].

The RecMap will include an adaptation feature, linking users to original evidence syntheses (e.g., summaries of benefits, harms, values, and impact on equity) and databases such as Epistemonikos for systematic reviews and primary studies. This feature will support users in contextualizing existing recommendations to different settings [34].

In another stage, we will identify and assess divergent recommendations, defined as scenarios in which two or more recommendations provide conflicting or differing courses of action for the same guideline question. We will examine divergence across four aspects including direction (for or against), strength (strong or conditional), certainty of the evidence (high, moderate, low, or very low) and contextual factors (values and preferences, cost/cost effectiveness, equity, acceptability, and feasibility). Using RecMap filters, reviewers will first confirm that compared recommendations match in terms of (1): Population, Intervention, Comparator, Outcome (PICO) elements with differing strength or direction, and (2) subgroup factors (e.g., dosage or timing differences). For fully aligned recommendations, two reviewers will independently conduct content analysis, combining deductive coding (based on RecMap themes) and inductive coding (to identify new themes). We will clearly classify divergences as methodological (e.g., different evidence bases or analytical approaches) or contextual (e.g., differing values and preferences or resources required). If divergences are methodological, we will highlight the more trustworthy recommendations. However, if they arise from contextual differences, we will transparently note them, allowing users to identify recommendations best suited to their settings.

#### 3.2.6 Early Integrated Knowledge Mobilization

Throughout the development phase of the e‐Chronic Pain RecMap, the project lead (AJD) with support from members of the steering committee will meet with our interest holders (Table 1) bi‐yearly via Zoom to provide progress updates and discuss challenges and potential solutions. Also, to facilitate patient‐healthcare practitioner shared decision making, we will develop, with the support of our patient partners, plain language recommendations and summaries (PLRs and PLSs), decision aids and infographics. Also, we will translate the English RecMap and its content into French, Spanish, and Mandarin. To ensure consistency in style and format, a translator (DM) will develop a translation style guide, glossary, and terminology references. These will guide collaboration with bilingual researchers, health professionals, and organizations to streamline the translation process for each language.

### 3.3 Phase 3: End of Project Knowledge Mobilization

We will conduct an overview of reviews of KM strategies used for guideline implementation across different levels of the healthcare system. Findings from our overview along with input from our experts and provincial, national, and international interest holders, will inform modifications of the strategies to disseminate the RecMap across Canada and internationally presented below.

End of project KM will entail planning, dissemination and knowledge exchange, and uptake and impact assessment. In terms of the planning stage, we will continue to engage key interest holders and knowledge users including, but not limited to, People living with pain groups ‐ including underserved or inequitably impacted populations (i.e., Migrants and Refugees, veterans, Indigenous Peoples, women, and children), public health agencies, researchers and guideline developers, educators, media, health care professionals, and policymakers. Our aim is to ensure collaborative planning and multi‐directional knowledge exchange with diverse interested individuals and groups including people with lived experience. Monthly virtual meetings will facilitate ongoing collaboration, track progress, and address challenges.

We will conduct an end‐of‐project dissemination plan based on the KM framework developed by Grimshaw et al. [35] and work by Lavis et al. [36] that includes: (1) publishing our findings in open‐access venues to target diverse audiences, including peer‐reviewed journals (health care providers, researchers), blogs and various social media outlets (patients and the public); (2) presenting our process and recommendations at regional and national conferences, and through press releases and interviews with news outlets; (3) sharing of PLRs, and developing decision aids, infographics, and educational videos in English, French, Spanish and Mandarin; and; (4) integration of the RecMap into educational modules, and organizational websites.

Finally, for uptake and impact assessment, we will track website analytics (e.g., user numbers, geographic locations, recommendation clicks) and assess barriers to RecMap use through interviews, focus groups, and surveys. Insights will inform content and functionality improvements to optimize accessibility and impact. Table 2 presents examples of unidirectional, bidirectional, and engagement knowledge mobilization activities and implementation strategies.

## 4 Discussion

The e‐Chronic Pain RecMap will synthesize all current guidelines on opioids, cannabis, and interventional spine procedures for chronic pain. We will tailor the presentation of materials to meet the needs of our three key groups including researchers, healthcare providers, and people with lived and living experience and their families. The map will provide users with knowledge to navigate decision‐making and address the variability and inconsistency of guideline recommendations for chronic pain management. Specifically, the RecMap will provide a platform for users to easily access trustworthy recommendations, compare recommendations addressing the same guideline question and evaluate reasons for divergence through trustworthiness classification and comparison of evidence and contextual factors.

### 4.1 Strengths and Limitations

The RecMap's strengths include identification of trustworthy, evidence‐based guidance through a centralized and intuitive platform. Its customizable design caters to diverse users, offering flexible viewing options such as recommendation lists, maps, and comparison tools. Equity‐sensitive filtering using frameworks such as PROGRESS‐PLUS supports the identification of context‐specific recommendations, while tools for contextualization allow guideline developers to adapt recommendations to local settings. The platform will promote transparency and trustworthiness by incorporating AGREE‐II and AGREE‐REX assessments, enabling users to note the quality of guidelines and recommendations. Also, it will facilitate identification of gaps and clusters in evidence, informing future research priorities and updates, while also supporting knowledge mobilization for improved clinical and health system decision‐making.

In terms of potential limitations, the utility of the RecMap will depend on the quality of included recommendations. Also, while equity‐sensitive features aim to address disparities, many guidelines may inadequately address marginalized populations. The map will highlight these gaps to guide future efforts in improving equity and trustworthiness. Additionally, the platform's extensive information risks overwhelming users without proper training or clear guidance. To mitigate this, feedback from focus groups and think‐aloud testing during development will inform usability improvements. Finally, maintaining a living RecMap is essential but resource‐intensive. Current resources enable updates and maintenance for the next 4 years, during which we will secure additional funding to ensure long‐term sustainability. Mitigating strategies will include (1) building capacity by integrating the effort into ongoing processes, such as engaging students and volunteers via Cochrane Exchange, social media, and McMaster initiatives, with certification for participation; (2) securing institutional support from the National Pain Center (NPC) at McMaster, which has a mandate to provide and maintain evidence‐based guidance for chronic pain management; (3) applying for follow‐up funding from CIHR to support continued maintenance, and (4) expanding the network, disseminating findings, conducting impact assessments, and pursuing additional donor and agency funding to sustain and grow the platform.

## 5 Conclusion

Our work will help limit the propagation of, and reliance on, untrustworthy guideline recommendations for management of chronic pain. Successful uptake will optimize evidence‐based management.

## Author Contributions

Conceptualization and Methodology: Andrea J. Darzi, Holger J. Schünemann, Jason W. Busse. Project Administration: Andrea J. Darzi, Kian Torabiardakani. Writing – Original Draft Preparation: Andrea J. Darzi. Writing – Review and Editing: Andrea J. Darzi, Kian Torabiardakani, Gonzalo Bravo‐Soto, Daniela Montalva‐Romero, Rachel J. Couban, Lynn Cooper, Stacey Ritz, Jaris Swidrovich, Ivan D. Florez, Vivian Welch, Gordon H. Guyatt, Holger J. Schünemann, Jason W. Busse.

## Conflicts of Interest

Andrea J. Darzi is the nominated principal investigator of this project and received CIHR funding through McMaster University to support this effort. Lynn Cooper is the Research and Education Director of the Canadian Injured Workers Alliance and supports with financial compensation the Power Over Pain Portal project at Ottawa Hospital Research Institute and ECHO Ontario. Jaris Swidrovich is on the Pain Ontario Board of Directors for the Pain Canada National Advisory Committee. Ivan D. Florez is the Editor in Chief of Clinical and Public Health Guidelines but did not participate in the editorial process of this article which was allocated to an ad hoc editor, and thus had no influence on the editorial decisions related to it. Holger Schünemann is the chair of the GRADE working group and has received funding for other Recommendation Map initiatives from CIHR. Recommendation Maps are programmed to GRADEpro which is the official software of the GRADE working group. None of the other authors have any conflicts of interest to disclose.

## Data Availability

All data associated with this protocol are available upon reasonable request from Andrea J. Darzi (darzia@mcmaster.ca).

## Appendix 1 Search strategies

Summary of search and strategy

**Table:**

MEDLINE	3858
Embase	7040
Subtotal	10898
‐dupes	−913
Total	9985

**MEDLINE**

**Database: OVID Medline Epub Ahead of Print, In‐Process & Other Non‐Indexed Citations, Ovid MEDLINE(R) Daily and Ovid MEDLINE(R) 1946 to July 18, 2024**

Search Strategy:

‐‐‐‐‐‐‐‐‐‐‐‐‐‐‐‐‐‐‐‐‐‐‐‐‐‐‐‐‐‐‐‐‐‐‐‐‐‐‐‐‐‐‐‐‐‐‐‐‐‐‐‐‐‐‐‐‐‐‐‐‐‐‐‐‐‐‐‐‐‐‐‐‐‐‐‐‐‐‐‐

1 (chronic adj4 pain*).mp. [mp=title, book title, abstract, original title, name of substance word, subject heading word, floating sub‐heading word, keyword heading word, organism supplementary concept word, protocol supplementary concept word, rare disease supplementary concept word, unique identifier, synonyms, population supplementary concept word, anatomy supplementary concept word] (97364)

2 Chronic Pain/ (24835)

3 exp Osteoarthritis/ (81327)

4 osteoarthrit*.mp. (120747)

5 osteo‐arthritis.mp. (403)

6 degenerative arthrit*.mp. (1472)

7 exp Arthritis, Rheumatoid/ (129419)

8 exp Neuralgia/ (25788)

9 Diabetic Neuropathies/ (16770)

10 (neuropath* adj5 (pain* or diabet*)).mp. (55412)

11 neuralg*.mp. (35368)

12 zoster.mp. (25215)

13 Irritable Bowel Syndrome/ (9972)

14 (IBS or irritable colon or irritable bowel).mp. [mp=title, book title, abstract, original title, name of substance word, subject heading word, floating sub‐heading word, keyword heading word, organism supplementary concept word, protocol supplementary concept word, rare disease supplementary concept word, unique identifier, synonyms, population supplementary concept word, anatomy supplementary concept word] (21650)

15 Migraine Disorders/ (30766)

16 migraine.mp. (47906)

17 Fibromyalgia/ (10384)

18 fibromyalg*.mp. (14910)

19 complex regional pain syndromes/or exp causalgia/or exp reflex sympathetic dystrophy/ (6060)

20 (complex regional pain syndromes or causalgia).mp. (2965)

21 Pain, Intractable/ (6431)

22 Phantom Limb/ (2128)

23 Hyperalgesia/ (14532)

24 ((noncancer* or non‐cancer* or cancer or malign* or chronic* or recurrent or persist* or non‐malign*) adj3 pain).mp. (112337)

25 or/1‐24 (533305)

26 exp back pain/or exp failed back surgery syndrome/or exp low back pain/ (46619)

27 Radiculopathy/or radiculopathy.mp. (11355)

28 musculoskeletal pain/or headache/ (37399)

29 exp Arthralgia/ (16365)

30 exp Headache Disorders/ (41633)

31 headache*.mp. (118368)

32 Temporomandibular Joint Dysfunction Syndrome/ (4960)

33 ((TMJ or TMJD) and pain*).mp. (3582)

34 whiplash.mp. or exp whiplash injury/ (4299)

35 exp Cumulative Trauma Disorders/ (15637)

36 exp Peripheral Nervous System Diseases/dt [Drug Therapy] (18388)

37 Pain Measurement/de [Drug Effects] (6924)

38 (backache* or backpain* or dorsalgi* or arthralgi* or polyarthralgi* or arthrodyni* or myalgi* or fibromyalgi* or myodyni* or neuralgi* or ischialgi* or crps or rachialgi*).ab,ti. (58539)

39 ((back or discogen* or bone or musculoskelet* or muscle* or skelet* or spinal or spine or vertebra* or joint* or arthritis or Intestin* or neuropath* or neck or cervical* or head or facial* or complex or radicular or cervicobrachi* or orofacial or somatic or shoulder* or knee* or hip or hips) adj3 pain).mp. (222400)

40 or/26‐39 (434887)

41 (acute or emergency or preoperative or postoperative).ti,ab. (2511813)

42 40 not 41 (359228)

43 25 or 42 (752814)

Annotation: chronic pain block from opioids review modified to include cancer pain

44 Multiple Sclerosis/ (65449)

45 Trigeminal Neuralgia/ (7910)

46 Parkinson Disease/ (86543)

47 Porphyrias/ (6744)

48 Endometriosis/ (26669)

49 Lupus Erythematosus, Systemic/ (63185)

50 Vulvodynia/ (600)

51 (functional adj3 disorder).mp. [mp=title, book title, abstract, original title, name of substance word, subject heading word, floating sub‐heading word, keyword heading word, organism supplementary concept word, protocol supplementary concept word, rare disease supplementary concept word, unique identifier, synonyms, population supplementary concept word, anatomy supplementary concept word] (5229)

52 Trigeminal Autonomic Cephalalgias/ (251)

53 Burning Mouth Syndrome/ (1177)

54 Anemia, Sickle Cell/ (24872)

55 Arthritis, Juvenile/ (12038)

56 (pain* or endometriosis or penoscrotodynia or vulvodynia or prostatits or sickle cell or cephalgia* or burning mouth or parkinson* or porphyria or lupus or sclerosis or MS or arthritis or arteriopathy or occlusive or metastat* or metastas* or polyneuropathy* or radiculopath* or endometriosis).mp. [mp=title, book title, abstract, original title, name of substance word, subject heading word, floating sub‐heading word, keyword heading word, organism supplementary concept word, protocol supplementary concept word, rare disease supplementary concept word, unique identifier, synonyms, population supplementary concept word, anatomy supplementary concept word] (2864531)

57 or/44‐56 (2874343)

Annotation: new pain* block

58 43 or 57 (3131526)

Annotation: sensitive pain concept

59 exp Analgesics, Opioid/ (140972)

60 (opioid* or opiate*).mp. [mp=title, book title, abstract, original title, name of substance word, subject heading word, floating sub‐heading word, keyword heading word, organism supplementary concept word, protocol supplementary concept word, rare disease supplementary concept word, unique identifier, synonyms, population supplementary concept word, anatomy supplementary concept word] (169494)

61 (alfentanil or alphaprodine or beta‐casomorphin$ or buprenorphine or carfentanil or codeine or deltorphin or dextromethorphan or dezocine or dihydrocodeine or dihydromorphine or enkephalin$ or ethylketocyclazocine or ethylmorphine or etorphine or fentanyl or heroin or hydrocodone or hydromorphone or ketobemidone or levorphanol or lofentanil or meperidine or meptazinol or methadone or methadyl acetate or morphine or nalbuphine or opium or oxycodone or oxymorphone or pentazocine or phenazocine or phenoperidine or pirinitramide or promedol or propoxyphene or remifentanil or sufentanil or tilidine or tapentadol).mp. [mp=title, book title, abstract, original title, name of substance word, subject heading word, floating sub‐heading word, keyword heading word, organism supplementary concept word, protocol supplementary concept word, rare disease supplementary concept word, unique identifier, synonyms, population supplementary concept word, anatomy supplementary concept word] (176645)

62 or/59‐61 (266331)

63 exp Narcotics/ (149250)

64 narcotic*.mp. (66808)

65 (adolonta or Anpec or Ardinex or Asimadoline or Alvimopam or amadol or biodalgic or biokanol or Codinovo or contramal or Demerol or Dicodid or Dihydrocodeinone or dihydromorphinone or dihydrohydroxycodeinone or dihydrone or dilaudid or dinarkon or dolsin or dolosal or dolin or dolantin or dolargan or dolcontral or duramorph or duromorph or duragesic or durogesic or eucodal or Fedotzine or Fentanest or Fentora or Fortral or Hycodan or Hycon or Hydrocodone or Hydrocodeinonebitartrate or hydromorphon or hydroxycodeinon or isocodeine or isonipecain or jutadol or laudacon or l dromoran or levodroman or levorphan or levo‐dromoran or levodromoran or lexir or lidol or lydol or morfin or morfine or morphia or morphin or morphinium or morphinene or morphium or ms contin or n‐methylmorphine or n methylmorphine or nobligan or numorphan or oramorph or oxycodeinon or oxiconum or oxycone or oxycontin or palladone or pancodine or pethidine or phentanyl or prontofort or robidone or skenan or sublimaze or sulfentanyl or sulfentanil or sufenta or takadol or talwin or theocodin or tramadol or tramadolhameln or tramadolor or tramadura or tramagetic or tramagit or tramake or tramal or tramex or tramundin or trasedal or theradol or tiral or topalgic or tradol or tradolpuren or tradonal or tralgiol or tramadorsch or tramadin or tramadoc or ultram or zamudol or zumalgic or zydol or zytram).mp. [mp=title, book title, abstract, original title, name of substance word, subject heading word, floating sub‐heading word, keyword heading word, organism supplementary concept word, protocol supplementary concept word, rare disease supplementary concept word, unique identifier, synonyms, population supplementary concept word, anatomy supplementary concept word] (13082)

66 or/59‐65 (299191)

Annotation: from opioids review

67 Cannabis/ (15072)

68 exp cannabinoids/or cannabidiol/or cannabinol/or dronabinol/ (19282)

69 Endocannabinoids/ (7320)

70 exp Receptors, Cannabinoid/ (11163)

71 (Cannabis or cannabinol or cannabinoid* or cannabidiol or bhang or cannador or charas or ganja or ganjah or hashish or hemp or marihuana or marijuana or nabilone or cesamet or cesametic or ajulemic acid or cannabichromene or cannabielsoin or cannabigerol or tetrahydrocannabinol or dronabinol or levonantradol or nabiximols or palmidrol or tetrahydrocannabinolic acid or tetrahydro cannabinol or marinol or tetranabinex or sativex or endocannabinoid*).mp. (76977)

72 or/67‐71 (76977)

Annotation: strategy from 2020 cannabis review

73 “marijuana use”/or marijuana smoking/ (7284)

74 Marijuana Abuse/ (7237)

75 (epidiolex or gwp 42003p or gwp42003p or nabidiolex or dronabinol or thc or tetrahydrocannabinol* or ea 1477 or ea1477 or marinol or qcd 84924 or syndros or tetrabinex or tetranabinex or cesamet or nabilone or deltanyne or “abbott 40566” or namisol or dronabinolum or “QCD 84924” or “CCRIS 4726” or nabiximol? or “gw 1000” or gw1000 or “sab 378” or sab378 or sativex).mp. [mp=title, book title, abstract, original title, name of substance word, subject heading word, floating sub‐heading word, keyword heading word, organism supplementary concept word, protocol supplementary concept word, rare disease supplementary concept word, unique identifier, synonyms, population supplementary concept word, anatomy supplementary concept word] (15950)

76 or/73‐75 (27824)

Annotation: cannabis terms from Wolfe 2020

77 or/67‐76 (78652)

78 injections/or injections, intra‐articular/or injections, spinal/or exp Denervation/or exp Nerve Block/or pulsed radiofrequency treatment/or radiofrequency ablation/or catheter ablation/or Radiography, Interventional/or Radiosurgery/ (219807)

79 exp Anesthetics, Local/ (113208)

80 exp Anesthesia, Conduction/ (73309)

81 exp glucocorticoids/or exp hydroxycorticosteroids/ (347689)

82 injection*.ti,ab. (662125)

83 ((epidural or interlaminar or transforaminal) adj3 (injection or injectate or space or block)).mp. [mp=title, book title, abstract, original title, name of substance word, subject heading word, floating sub‐heading word, keyword heading word, organism supplementary concept word, protocol supplementary concept word, rare disease supplementary concept word, unique identifier, synonyms, population supplementary concept word, anatomy supplementary concept word] (12440)

84 (Adhesiolysis or denervation* or epiduroscopy or neurotomy).mp. [mp=title, book title, abstract, original title, name of substance word, subject heading word, floating sub‐heading word, keyword heading word, organism supplementary concept word, protocol supplementary concept word, rare disease supplementary concept word, unique identifier, synonyms, population supplementary concept word, anatomy supplementary concept word] (39077)

85 (hypertonic saline neurolysis or Epidural lysis or Epidural endoscopy or Racz or spinal endoscopy or epidural adhesion*).tw. (336)

86 ((Epidural or peridural or lumbar or spinal or low back) and (neuroplasty or neurolysis or epidurolysis or lysis)).tw. (791)

87 (local anaesthetic or epidural steroid or bupivacaine or carbocain or corticosteroid* or dexamethasone or glucocorticoid* or hyaluronoglucosaminidase or Hyaluronidase or lidocaine or mepivacaine or methylprednisolone).mp. [mp=title, book title, abstract, original title, name of substance word, subject heading word, floating sub‐heading word, keyword heading word, organism supplementary concept word, protocol supplementary concept word, rare disease supplementary concept word, unique identifier, synonyms, population supplementary concept word, anatomy supplementary concept word] (384010)

88 or/78‐87 (1455247)

Annotation: from interventional procedures review

89 66 or 77 or 88 (1761804)

Annotation: sensitive interventions concept

90 58 and 89 (329809)

91 guideline.pt. (16387)

92 consensus/ (23240)

93 exp consensus development conferences as topic/ (3010)

94 exp Guidelines as Topic/ (174425)

Annotation: explode includes practice guidelines

95 Guidelines as Topic/ (42233)

Annotation: no explode, does not include practice guidelines

96 Health Planning Guidelines/ (4165)

97 consensus development conference.pt. (12464)

98 position statement?.ti. (4147)

99 policy statement?.ti. (1029)

100 practice parameter?.ti. (708)

101 guideline*.ti. (98774)

102 consensus.ti. (35321)

103 scientific statement?.ti. (822)

104 performance measure?.ti. (1512)

105 recommendation?.ti. (53384)

106 or/91‐105 (329066)

107 (comment or interview or letter or news or case report).pt. (2016628)

108 106 not 107 (298446)

109 limit 108 to yr = “2019 ‐Current” (72367)

110 90 and 108 (3858)

**Embase**

**Database: Embase <1974 to 2024 July 17>**

**Search Strategy:**

‐‐‐‐‐‐‐‐‐‐‐‐‐‐‐‐‐‐‐‐‐‐‐‐‐‐‐‐‐‐‐‐‐‐‐‐‐‐‐‐‐‐‐‐‐‐‐‐‐‐‐‐‐‐‐‐‐‐‐‐‐‐‐‐‐‐‐‐‐‐‐‐‐‐‐‐‐‐‐‐

1 (chronic adj4 pain*).mp. [mp=title, abstract, heading word, drug trade name, original title, device manufacturer, drug manufacturer, device trade name, keyword heading word, floating subheading word, candidate term word] (157668)

2 chronic pain/ (82773)

3 exp osteoarthritis/ (170436)

4 osteoarthrit*.mp. (192290)

5 osteo‐arthritis.mp. (501)

6 degenerative arthrit*.mp. (1780)

7 exp rheumatoid arthritis/ (248834)

8 exp neuralgia/ (132106)

9 diabetic neuropathy/ (30085)

10 (neuropath* adj5 (pain* or diabet*)).mp. (99507)

11 neuralg*.mp. (38145)

12 zoster.mp. (49057)

13 irritable colon/ (33911)

14 (Irritable Bowel Syndrome or IBS).mp. [mp=title, abstract, heading word, drug trade name, original title, device manufacturer, drug manufacturer, device trade name, keyword heading word, floating subheading word, candidate term word] (33126)

15 exp migraine/ (82237)

16 migraine.mp. (90595)

17 fibromyalgia/ (26870)

18 fibromyalg*.mp. (29046)

19 reflex sympathetic dystrophy.mp. (2431)

20 (complex regional pain syndromes or causalgia).mp. (1400)

21 intractable pain/ (5737)

22 phantom limb.mp. or agnosia/or phantom pain/or amputation stump/ (8957)

23 hyperalgesia/ (22537)

24 ((noncancer* or non‐cancer* or cancer or malign* or chronic* or recurrent or persist* or non‐malign*) adj3 pain).mp. (206951)

25 or/1‐24 (967398)

26 exp backache/ (143986)

27 radiculopathy.mp. or exp radiculopathy/ (50567)

28 musculoskeletal pain/ (16424)

29 exp arthralgia/ (84215)

30 headache/ (290803)

31 headache*.mp. (366837)

32 temporomandibular joint disorder/ (16880)

33 ((TMJ or TMJD) and pain*).mp. (4824)

34 whiplash.mp. or whiplash injury/ (5505)

35 exp cumulative trauma disorder/ (25874)

36 or/26‐35 (642321)

37 (acute or emergency or preoperative or postoperative).ti,ab. (3493491)

38 36 not 37 (528647)

39 25 or 38 (1352413)

40 multiple sclerosis/ (164632)

41 trigeminus neuralgia/ (13155)

42 Parkinson disease/ (201576)

43 porphyria/ (4397)

44 endometriosis/ (48603)

45 systemic lupus erythematosus/ (116957)

46 vulvodynia/ (2070)

47 (functional adj3 disorder).mp. [mp=title, abstract, heading word, drug trade name, original title, device manufacturer, drug manufacturer, device trade name, keyword heading word, floating subheading word, candidate term word] (8345)

48 trigeminal autonomic cephalalgia/ (813)

49 burning mouth syndrome/ (2062)

50 sickle cell anemia/ (44897)

51 juvenile rheumatoid arthritis/ (25009)

52 (pain* or endometriosis or penoscrotodynia or vulvodynia or prostatits or sickle cell or cephalgia* or burning mouth or parkinson* or porphyria or lupus or sclerosis or MS or arthritis or arteriopathy or occlusive or metastat* or metastas* or polyneuropathy* or radiculopath* or endometriosis).mp. (4556163)

53 or/40‐52 (4566577)

54 39 or 53 (5030357)

Annotation: sensitive pain concept

55 exp narcotic analgesic agent/ (422980)

56 (opioid* or opiate*).mp. [mp=title, abstract, heading word, drug trade name, original title, device manufacturer, drug manufacturer, device trade name, keyword heading word, floating subheading word, candidate term word] (258988)

57 (alfentanil or alphaprodine or beta‐casomorphin$ or buprenorphine or carfentanil or codeine or deltorphin or dextromethorphan or dezocine or dihydrocodeine or dihydromorphine or enkephalin$ or ethylketocyclazocine or ethylmorphine or etorphine or fentanyl or heroin or hydrocodone or hydromorphone or ketobemidone or levorphanol or lofentanil or meperidine or meptazinol or methadone or methadyl acetate or morphine or nalbuphine or opium or oxycodone or oxymorphone or pentazocine or phenazocine or phenoperidine or pirinitramide or promedol or propoxyphene or remifentanil or sufentanil or tilidine or tapentadol).mp. (354685)

58 (adolonta or Anpec or Ardinex or Asimadoline or Alvimopam or amadol or biodalgic or biokanol or Codinovo or contramal or Demerol or Dicodid or Dihydrocodeinone or dihydromorphinone or dihydrohydroxycodeinone or dihydrone or dilaudid or dinarkon or dolsin or dolosal or dolin or dolantin or dolargan or dolcontral or duramorph or duromorph or duragesic or durogesic or eucodal or Fedotzine or Fentanest or Fentora or Fortral or Hycodan or Hycon or Hydrocodone or Hydrocodeinonebitartrate or hydromorphon or hydroxycodeinon or isocodeine or isonipecain or jutadol or laudacon or l dromoran or levodroman or levorphan or levo‐dromoran or levodromoran or lexir or lidol or lydol or morfin or morfine or morphia or morphin or morphinium or morphinene or morphium or ms contin or n‐methylmorphine or n methylmorphine or nobligan or numorphan or oramorph or oxycodeinon or oxiconum or oxycone or oxycontin or palladone or pancodine or pethidine or phentanyl or prontofort or robidone or skenan or sublimaze or sulfentanyl or sulfentanil or sufenta or takadol or talwin or theocodin or tramadol or tramadolhameln or tramadolor or tramadura or tramagetic or tramagit or tramake or tramal or tramex or tramundin or trasedal or theradol or tiral or topalgic or tradol or tradolpuren or tradonal or tralgiol or tramadorsch or tramadin or tramadoc or ultram or zamudol or zumalgic or zydol or zytram).mp. [mp=title, abstract, heading word, drug trade name, original title, device manufacturer, drug manufacturer, device trade name, keyword heading word, floating subheading word, candidate term word] (65483)

59 or/55‐58 (530251)

60 cannabis/ (47237)

61 medical cannabis/ (4522)

62 exp cannabinoid receptor/ (18353)

63 cannabis addiction/ (12255)

64 “cannabis use”/or cannabis smoking/ (20148)

65 exp cannabinoid/ (93136)

66 (Cannabis or cannabinol or cannabinoid* or cannabidiol or bhang or cannador or charas or ganja or ganjah or hashish or hemp or marihuana or marijuana or nabilone or cesamet or cesametic or ajulemic acid or cannabichromene or cannabielsoin or cannabigerol or tetrahydrocannabinol or dronabinol or levonantradol or nabiximols or palmidrol or tetrahydrocannabinolic acid or tetrahydro cannabinol or marinol or tetranabinex or sativex or endocannabinoid*).mp. (123004)

67 or/60‐66 (124675)

Annotation: cannabis concept

68 denervation/or exp neurotomy/or exp nerve block/or exp catheter ablation/or radiofrequency ablation/or radiofrequency catheter ablation/or radiofrequency therapy/or pulsed radiofrequency treatment/or interventional radiology/or nonsurgical invasive therapy/or exp radiosurgery/or neurolysis/or rhizotomy/ (233779)

69 (Adhesiolysis or denervation* or epiduroscopy or neurotomy or neurolysis or rhizotomy or epidurolysis or neuroplasty or lysis or thermocoag* or electrocoag*).ti,ab. (116588)

70 ((nerve or radiofrequency or radio‐frequency or RF) adj3 (ablation or ablative)).mp. (65801)

71 ((lateral or medial or nerve) adj5 block).mp. (49168)

72 or/68‐71 (343997)

73 injection/or exp intraspinal drug administration/or intraarticular drug administration/ (172774)

74 regional anesthesia/or exp nerve block/ (71158)

75 (injection* or inject* or infus* or infiltrate* or intravenous* or intra venous or intramuscular* or intra muscular or intradisc* or intradisk* or IV or IM).ti,ab. (2627791)

76 or/73‐75 (2703042)

Annotation: injection

77 exp local anesthetic agent/or exp corticosteroid/or hyaluronic acid/ (1487432)

78 (local anaesthetic or local anasthetic or epidural steroid or corticosteroid* or glucocorticoid* or dexamethasone or triamcinolone or betamethasone or methylprednisolone or bupivacaine or carbocain or Lidocaine or xylocaine or Levobupivacaine or Mepivacaine or Prilocaine or Hydrocortisone or prednisolone or Prednisone or Depo‐medrol or Solu‐medrol or Amvisc or Healon or Hyalgan or hyaluronan or hyaluronate or hyaluronic or Hylaform or hylan or orthovisc or Synvisc or hyaluronoglucosaminidase or Hyaluronidase).ti,ab. (614587)

79 77 or 78 (1574921)

Annotation: drugs of interest

80 76 and 79 (290710)

Annotation: injection AND drugs of interest

81 59 or 67 or 72 or 80 (1178358)

Annotation: all interventions

82 54 and 81 (392611)

83 exp consensus/ (107454)

84 exp practice guideline/ (756271)

85 health care planning/ (112954)

86 position statement?.ti. (4558)

87 policy statement?.ti. (1130)

88 practice parameter?.ti. (830)

89 guideline*.ti. (132389)

90 consensus.ti. (43265)

91 scientific statement?.ti. (904)

92 performance measure?.ti. (1890)

93 recommendation?.ti. (66970)

94 or/83‐93 (1046001)

95 letter. pt. (1330366)

96 94 not 95 (994959)

97 82 and 96 (17702)

98 limit 97 to yr = “2019 ‐Current” (7040)
